# Tissue-specific gene expression and protein abundance patterns are associated with fractionation bias in maize

**DOI:** 10.1186/s12870-019-2218-8

**Published:** 2020-01-03

**Authors:** Jesse R. Walsh, Margaret R. Woodhouse, Carson M. Andorf, Taner Z. Sen

**Affiliations:** 1 U.S. Department of Agriculture, Agricultural Research Service, Corn Insects and Crop Genetics Research Unit, Ames, IA 50011 USA; 2 Department of Ecology, Evolution, and Organismal Biology, Iowa State University, Ames, IA 50011 USA; 3 U.S. Department of Agriculture, Agricultural Research Service, Western Regional Research Center, Crop Improvement and Genetics Research Unit, Albany, CA 94710 USA; 4 Department of Computer Science, Iowa State University, Ames, IA 50011 USA; 5 Department of Genetics, Development, and Cell Biology, Iowa State University, Ames, IA 50011 USA

**Keywords:** Subgenome, Gene expression, Protein abundance, Maize, Functional divergence

## Abstract

**Background:**

Maize experienced a whole-genome duplication event approximately 5 to 12 million years ago. Because this event occurred after speciation from sorghum, the pre-duplication subgenomes can be partially reconstructed by mapping syntenic regions to the sorghum chromosomes. During evolution, maize has had uneven gene loss between each ancient subgenome. Fractionation and divergence between these genomes continue today, constantly changing genetic make-up and phenotypes and influencing agronomic traits.

**Results:**

Here we regenerate the subgenome reconstructions for the most recent maize reference genome assembly. Based on both expression and abundance data for homeologous gene pairs across multiple tissues, we observed functional divergence of genes across subgenomes. Although the genes in the larger maize subgenome are often expressing more highly than their homeologs in the smaller subgenome, we observed cases where homeolog expression dominance switches in different tissues. We demonstrate for the first time that protein abundances are higher in the larger subgenome, but they also show tissue-specific dominance, a pattern similar to RNA expression dominance. We also find that pollen expression is uniquely decoupled from protein abundance.

**Conclusion:**

Our study shows that the larger subgenome has a greater range of functional assignments and that there is a relative lack of overlap between the subgenomes in terms of gene functions than would be suggested by similar patterns of gene expression and protein abundance. Our study also revealed that some reactions are catalyzed uniquely by the larger and smaller subgenomes. The tissue-specific, nonequivalent expression-level dominance pattern observed here implies a change in regulatory control which favors differentiated selective pressure on the retained duplicates leading to eventual change in gene functions.

## Background

Whole-genome duplication through polyploidy has occurred in many eukaryotes, including wheat, soybean, and cotton [1]. By some estimates, between 50 and 95% of all angiosperms have experienced at least one polyploidy event [2, 3]. Maize is believed to have diverged from sorghum during an ancient speciation event [4], followed by a whole-genome duplication event which occurred between 5 and 12 million years ago, causing the progenitor of maize to become tetraploid [5]. After genome duplication and subsequent fusion, maize is reduced back to a diploid state [6]. Gene loss occurred in one copy of the genome at a greater rate than the other copy--a phenomenon called fractionation bias acting through a short deletion mechanism [7], resulting in the larger (Maize1) and smaller (Maize2) subgenomes. Previous work by Brohammer et al. [8] has found that fractionation patterns appear similar between multiple maize lines.

Functional divergence of duplicated genes [9] has previously been studied in Arabidopsis [10], Brassica [11], cotton [2] and maize [12]. It is presumed that the accumulation of single nucleotide polymorphisms (SNPs) and insertions/deletions (INDELs) as well as changes to upstream regulatory elements and transposon interference cause functional divergence. A study of Pol IV-mediated gene silencing in maize [13] found transposable elements (TE) do affect expression and are (for rpd1/rmr6) more common on Maize2 than Maize1, while non-syntenic genes are more likely to have TEs upstream (i.e. likely created by TE shuffling).

Previous work by Moore et al. [14] illustrates some of the possible fates duplicated genes may experience given enough evolutionary time. While we cannot compare modern genes directly to their ancient counterparts, we can derive insight into the possible outcomes experienced over evolutionary time by comparing modern duplicates to each other. If we assume that the whole-genome duplication event resulted in duplicate genes with similar functions and expression patterns, it stands to reason that changes in expression pattern between conserved duplicated genes will have resulted in either a gain or loss of regulatory function in one of the copies.

The relationship between the expression of retained duplicates is generally described as favoring the dominant (Maize1) subgenome [9, 15, 16] in aggregate; however, there is evidence that the relationship between retained duplicates is not always a simple dominance relationship [16]. In cotton, there is evidence of reciprocal silencing [2] such that one copy in a retained duplicate pair expresses in some tissues but not others, while the other copy expresses in a different set of tissues. Previous analysis of expression data [16] showed a prevailing trend for the Maize1 genome to express at a higher rate than the Maize2 genome on average, but also identified what were described as retained duplicates in which duplicate genes switched dominance in different tissues. Furthermore, they found that repressed genes tend to experience greater transposon load, but do not differentiate significantly in distance from the 5′ end of the open reading frame to the nearest upstream transposon when compared to non-repressed genes.

In this work, we regenerate the subgenome assignments using a previously described protocol [15] while using the most recent maize reference genome assembly, B73 RefGen_v4 [17]. By looking at gene expression and protein abundance patterns from an existing expression atlas [18] we investigate functional differences between the homeologous copies. We additionally look at gene ontology [19, 20] annotations from MaizeCyc [21] and UniProt [22], Pathways from CornCyc [23], and the number of isoforms per gene model in the structural annotations provided by the genome assembly group [24]. We find that the Maize1 subgenome has a greater range of GO assignments, and that there is a relative lack of overlap between the subgenomes in terms of GO annotations than would be suggested by expression and abundance data. We demonstrate for the first time that protein abundances are higher in Maize1 than Maize2, but they also show tissue-specific dominance, a pattern similar to RNA expression dominance in Maize1. We also find that pollen expression is uniquely decoupled from protein abundance. Finally, we suggest a method for categorizing retained pairs based on expression patterns.

## Results

### Subgenome assignments in RefGen_v4

Synonymous mutations are generally thought to be under weak selective constraint and thus accumulate gradually over time. This premise can be exploited to estimate regions of similar age by comparing the Ks (synonymous mutation rate) of regions of synteny to an outgroup. We followed the methodology previously described in Schnable et al. [15] to generate subgenome assignments for gene models in B73 RefGen_v4 using the following modifications. Syntenic regions between maize and sorghum were determined by comparing the unmasked maize B73 RefGen_v4 against the unmasked *Sorghum bicolor* v3.1 within the SynMap tool hosted on CoGe [25]. We filtered out small syntenic blocks containing less than 12 genes and syntenic blocks with a block average Ks higher than 1.0. We utilized a greedy approach to group syntenic blocks by size with non-overlapping gene models relative to sorghum.

A recent study suggests a common evolutionary relationship between maize and current day Urelytrum/Vossia which may provide clues to the nature of the parents of the allopolyploidy event that followed the speciation of ancestral maize and ancestral sorghum [26]. It has been suggested that these parents could be linked to the modern subgenomes in maize such that the origins of the subgenomes could be revealed, however for the purposes of this study we continue to use the definitions of Maize1 and Maize2 referred to in Schnable et al. [15]. We assigned the larger, less fractionated set to the Maize1 subgenome and the Smaller, more fractionated set to the Maize2 subgenome. Additionally, it is possible that codon bias and violations of the assumption of weak selective constraints for synonymous mutations, which have been observed previously in other organisms [27, 28], may affect how subgenome assignments are performed, however we are not currently aware of a benchmark for accumulation of silent mutations which would allow for correction of this possibility. A list of subgenome assignments and retained duplicate genes are available in Additional file 1.

### Dominant homeologs are dispersed throughout each genome

**Fig. 1:**
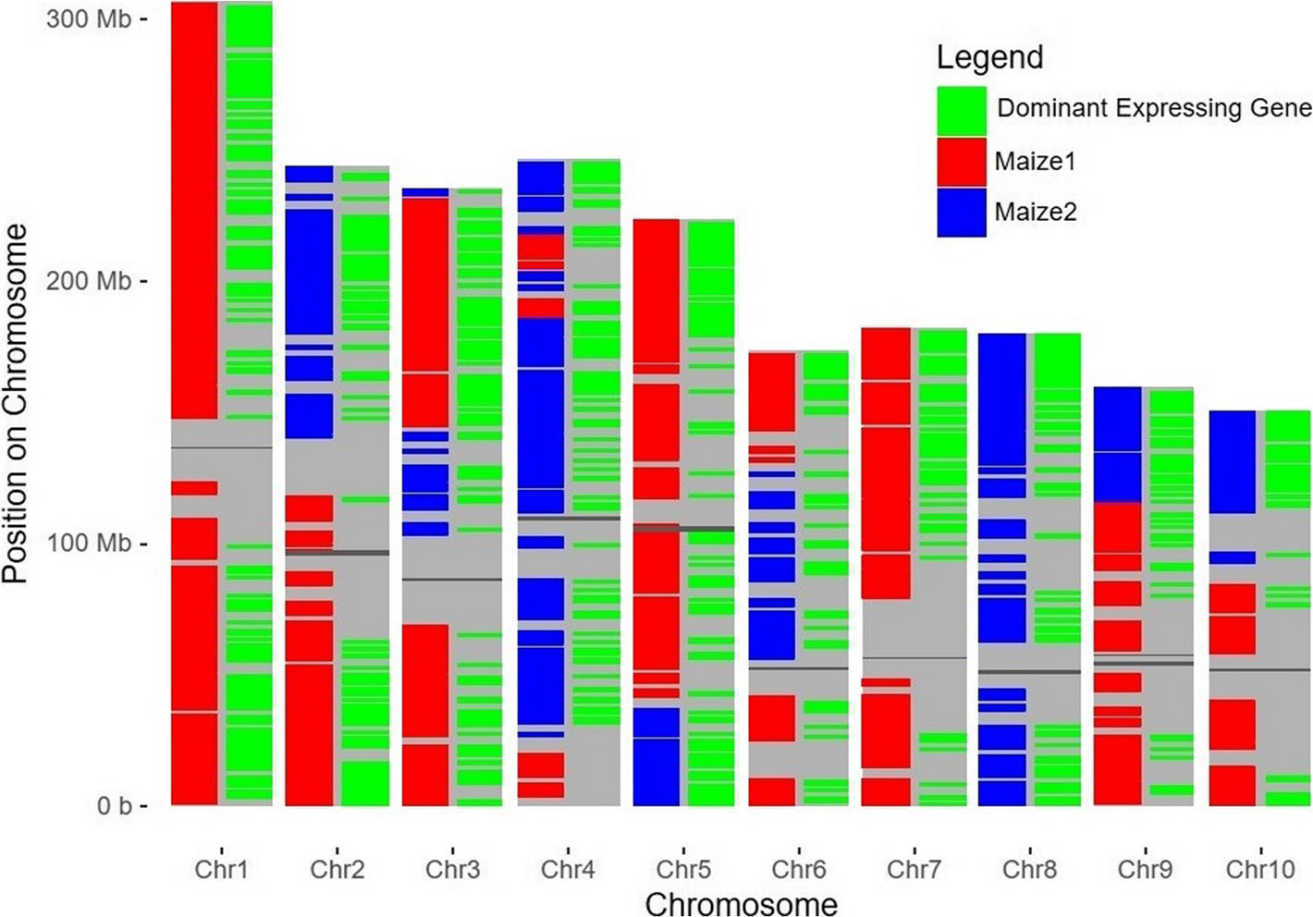
Identified subgenomes mapped to maize chromosomes to demonstrate dominant expression for homeologous gene pairs. Each chromosome has 2 columns. A grey line represents the centromere for each chromosome. The left column represents gene models assigned to either Maize1 (red) or Maize2 (blue). In the right column for each chromosome, a green line represents a gene that dominates its homeolog in gene expression by at least 2-fold, averaged across all tissues. There are 831 pairs where the Maize1 gene expressed 2-fold higher, 528 pairs where the Maize2 gene expressed 2-fold higher, and 1905 pairs where neither gene was 2-fold higher than the other

The locations of Maize1 and Maize2 are plotted on their chromosomes in Fig. 1. A retained duplicate that over-expressed its copy by at least 2-fold on average across all tissues is marked on the right of each chromosome (see Additional file 2). Such cases of dominance by one or the other copy are widely distributed across all chromosomes. The distal end of chromosome 4 assigned to Maize1 was noticeably absent of dominant retained duplicates.

### Gene expression and protein abundance are more often greater in Maize1, but not always in the same tissue and at the same growth level

Gene expression was measured in fragments per kilobase of transcript per million FPKM using STAR [29] and Cufflinks [30] on sequencing data available for B73 [18]. Cufflinks distributes reads that map to multiple loci evenly between those locations, which allows it to deal with ambiguities that arise from reads that map to multiple homeologs. Maize1 homeologs are observed to express 2-fold or higher than their duplicate Maize2 homeologs when averaged across all tissues. For each tissue, between 51.1 and 57.9% of Maize1 genes express higher than those of Maize2, except in two cases: the average Maize2 gene expression in FPKMs was much greater than Maize1 for endosperm crown 27 days after planting (DAP) and somewhat higher in mature pollen and pericarp 27 DAP.

We find that Maize1 homeolog are also observed to have 2-fold or greater protein abundances when compared to their Maize2 homeolog on average. The average protein abundance in mature pollen was particularly high in both subgenomes relative to the other tissues and much greater in Maize1 relative to Maize2. But the average Maize2 protein abundance in dNSAF (distributed Normalized Spectral Abundance Factor) [31] was greater in 7 tissues, including mature leaf, root meristem, root elongation, endosperm 12 DAP, secondary root, primary root, and vegetative meristem.

**Fig. 2:**
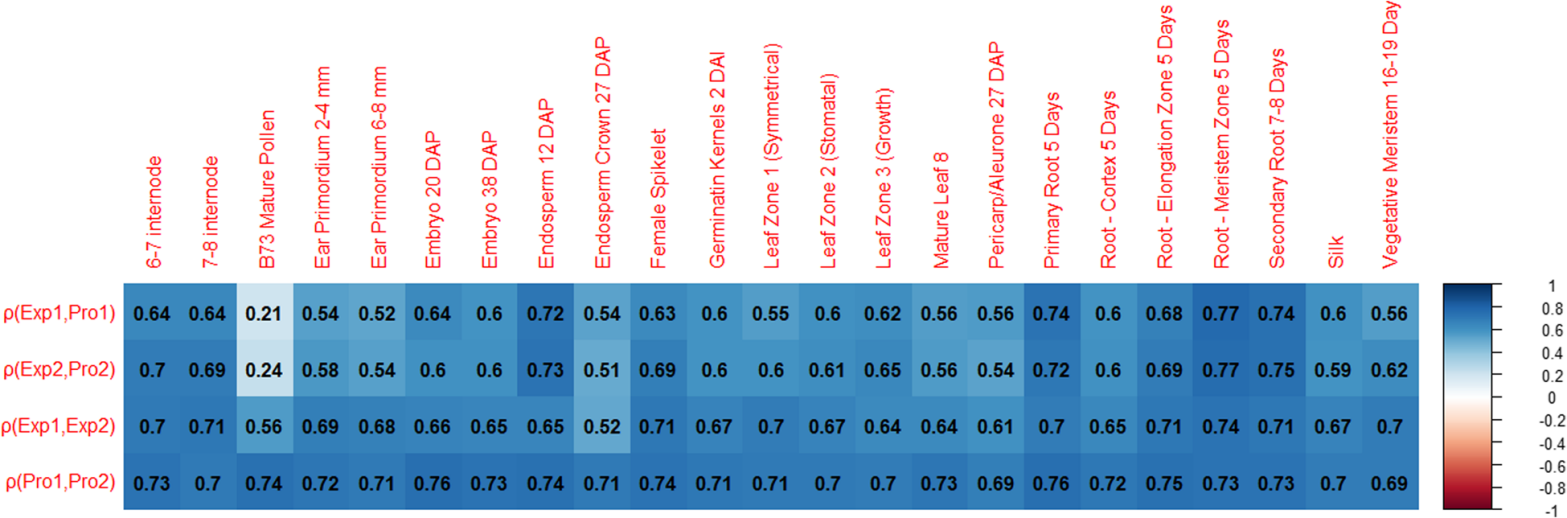
Spearman correlation matrix for RNA expression and protein abundance for the 23 tissues captured in this dataset. Moderate positive correlation was found between RNA expression and protein abundance for genes in Maize1 (row 1) and Maize2 (row 2) for all tissues except B73 mature pollen. For mature pollen, Maize1 expression was weakly correlated to protein abundance with *ρ* = 0.2060438 (*p*-value = 2.866e-05) and Maize2 expression was weakly correlated to protein abundance with ρ = 0.2354009 (*p*-value = 4.061e-06). The Maize1 homeologs are moderately correlated to Maize2 homeologs for both expression (row 3) and protein abundance in each tissue (row 4)

B73 mature pollen was found to have fewer expressed genes than other tissues as well as fewer detectable protein abundances. Pollen had the lowest correlation between expression and abundance (Fig. 2) and contained several genes with very high expression. Previous studies in other maize datasets found pollen to have higher expression of transcription factors in general and to have higher expression of genes from Maize2 [32]. While we find that the Maize1 gene expresses higher than its Maize2 counterpart more often than the reverse, the average FPKM for genes in Maize2 are higher than in Maize1 for B73 mature pollen and endosperm crown 27 DAP. Furthermore, if the FPKM of all genes (not just those with a retained duplicate) from each subgenome is averaged, only 5 of the 23 tissues have higher average expression in Maize1 vs. Maize2.

### Expression and abundance show tissue-specific dominant patterns for retained duplicates

**Fig. 3:**
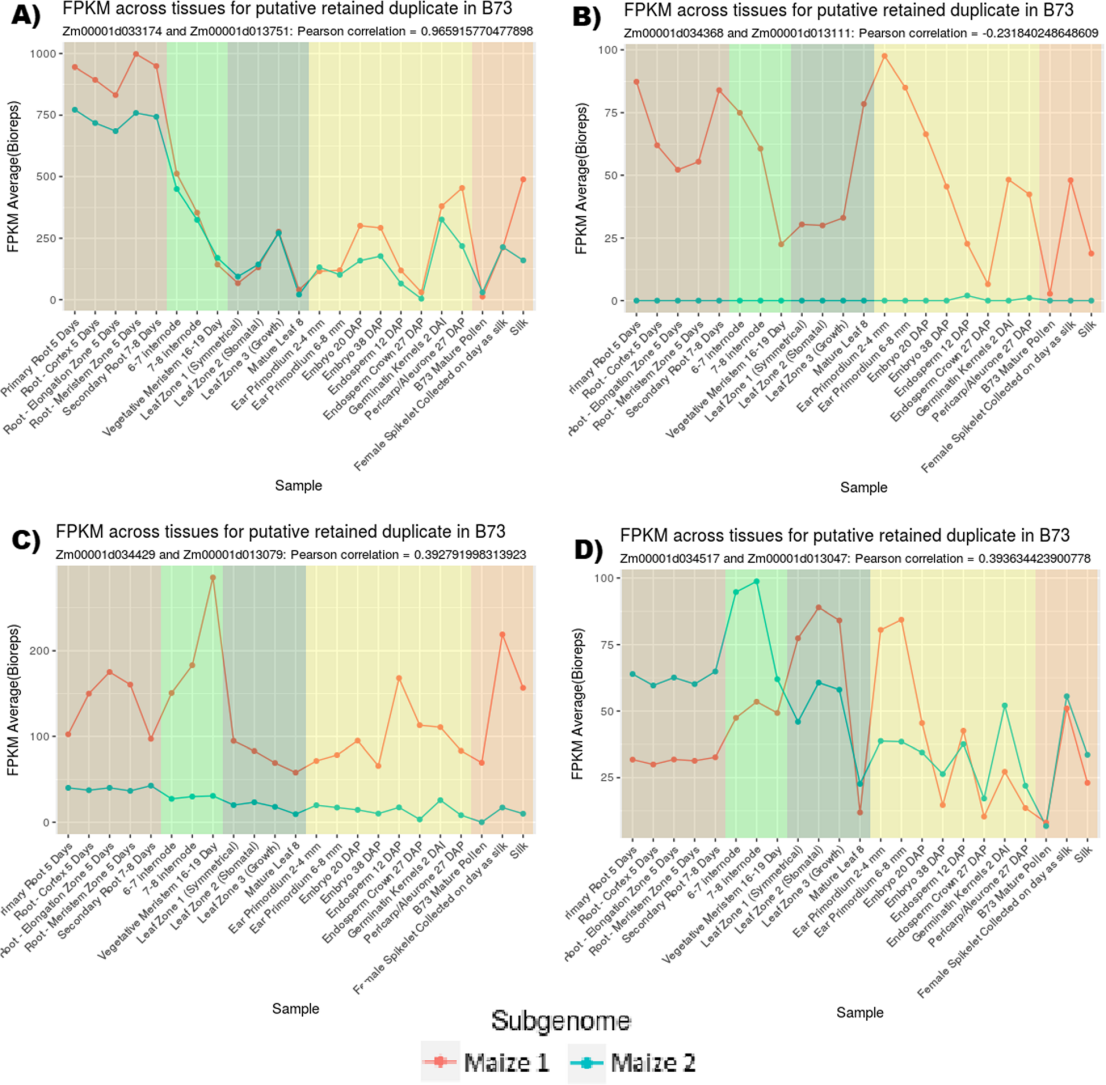
Tissue-specific expression dominance patterns. While each pattern is distinct, averaging FPKM across tissues would mask these patterns. Tissues are grouped roughly by type: brown for roots, light green for stalk, dark green for leafy tissue, yellow for kernel, and orange for reproductive. Each pattern shows a different characteristic: **a** highly correlated expression with an average dominance of one homeolog, **b** one homeolog has little or no detected expression, **c** one homeolog expresses much less than the other under all available tissues, and **d** both homeologs are the dominant expresser under different conditions

Analysis of gene expression often uses averages or other aggregates to determine fold change and expression dominance. However, larger trends in expression dominance between retained duplicates can mask individual nuance found when examining patterns of expression across different tissues, conditions, or time points. Fig. 3 presents four representative cases in which one of the retained duplicates can be said to be the “dominant” homeolog in terms of expression when looking only at average FPKM across available tissues. These scenarios include but are not limited to: A) highly correlated expression with an average dominance of one homeolog, B) one homeolog has little or no detected expression, C) one homeolog expresses much less than the other under all available tissues, and D) both homeologs are the dominant expresser under different conditions, yet one homeolog still has a higher averaged expression.

Of the 3660 retained duplicate pairs in our study, we find 37 pairs where both genes had no detected expression across all 23 tissues. There were 274 pairs where one or the other gene had no detectable expression, but not both. A set of 930 pairs (25% of the pairs) exhibited alternating dominance, where both genes had at least 1 tissue in which they expressed 2-fold higher than their homeolog.

Of the remaining retained duplicate pairs, 265 had a correlation > 0.95. The remaining 2154 pairs did not fit these target patterns. These pairs are all expressed in at least one tissue for both genes and do not exhibit clear dominance nor do they have high positive correlation. There are several possible scenarios which might describe the function of these gene pairs. One possibility is that they are selectively constrained through a dosage compensation which prevented fractionation of one or both genes. Another possibility is that these genes diverged functionally in a way that is not clearly revealed through their expression patterns. Further investigation may serve to differentiate these cases.

A similar analysis was performed using the protein abundance data. We find 8 pairs where both proteins had no detected abundance across all 23 tissues. There were 133 pairs where one protein of a pair had detectable abundance and the other did not. A set of 353 (10%) of the pairs exhibited alternating dominance, where both proteins had at least 1 tissue in which they had 2-fold higher abundance than their homeolog. Of the remaining pairs, 98 pairs had a correlation > 0.95. The remaining 3068 pairs did not fit these patterns.

For 1372 retained duplicate pairs, there is both expression in at least 1 of the 23 tissues and abundance measurements in at least 1 of the 23 tissues for both genes (referred to as “DataComplete” in Additional file 2). A total of 122 (9%) of these pairs exhibited alternating dominance, where both genes had at least 1 tissue in which they expressed 2-fold higher than their homeolog and had protein abundance 2-fold higher than their homeolog. Of the remaining pairs, 50 pairs had a correlation > 0.95 in both their expression and abundance.

### Functional annotations imply greater diversity in Maize1

**Fig. 4:**
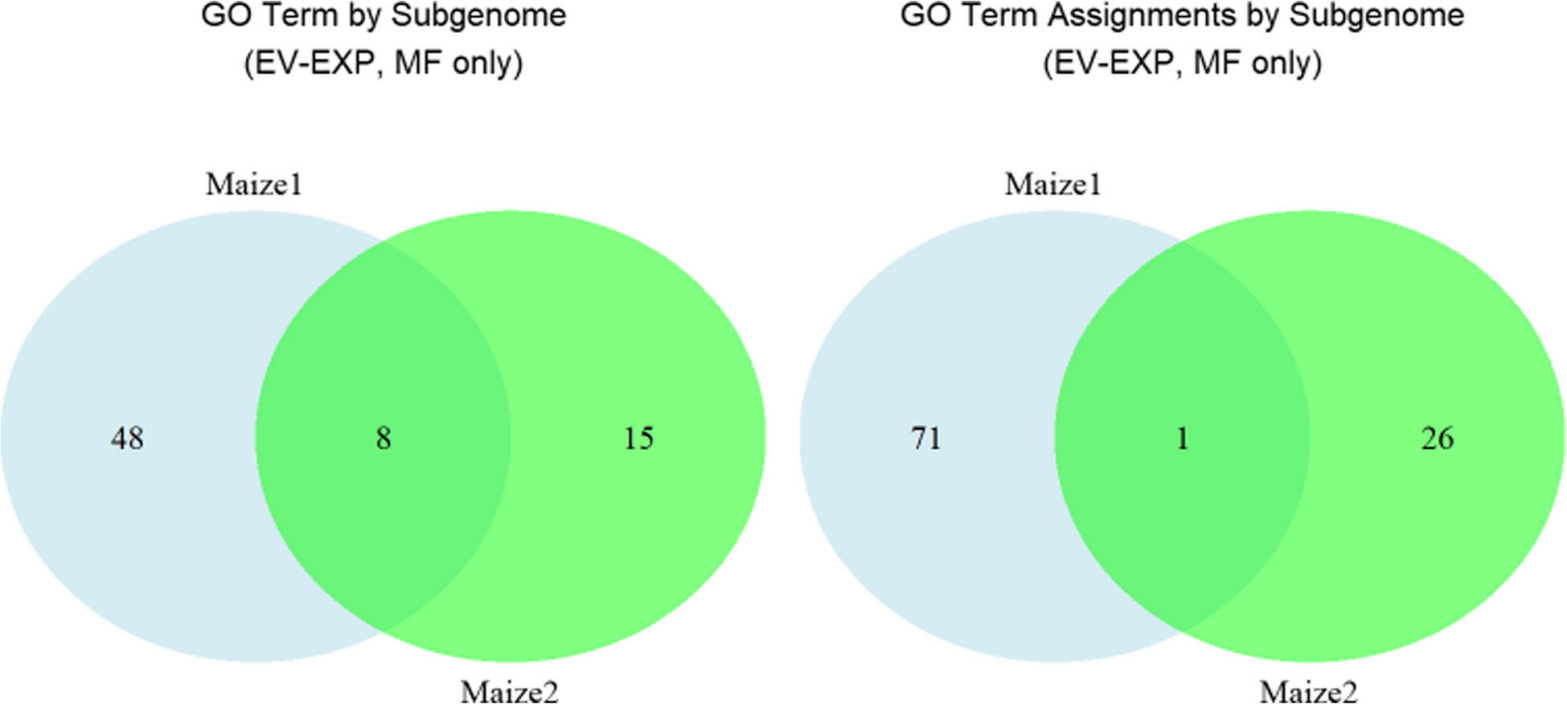
(Left) Number of unique GO terms annotated by at least one gene in the subgenome set of genes. Taken as a whole, there are more GO experimentally determined molecular function (MF) type GO terms annotated to Maize1 than to Maize2. (Right) When considering GO terms annotated to homeolog pairs, Maize1 has more annotations than Maize2. Certain GO terms are annotated to several genes, which is why there are more total annotations than GO terms present. Also, some cases exist where the same GO term is annotated to one of the homeolog pairs in one subgenome but a different (non-homeolog) gene in the other subgenome. Such cases could be due to a need for additional manual curation or might imply a loss-of-function and/or gain-of-function event

A comparison of GO annotations assigned to genes in Maize1 and Maize2 reveals a greater range of GO terms in Maize1. When considering both computationally assigned and manually curated GO annotations, we find 1254 GO terms present for genes in both Maize1 and Maize2, with Maize1 having 599 terms unique to Maize1 and Maize2 having 158 unique terms (see Additional file 3). GO terms manually assigned to genes using experimentally-determined evidence codes are considered the gold standard of annotation assignment. Comparing only these gold standard GO annotations between the subgenomes, we find 8 GO terms present for genes in both Maize1 and Maize2, while Maize1 has 48 unique terms and Maize2 has 15 unique terms shown in Fig. 4 (left). Considering each GO assignment to an individual gene, Maize1 has 71 unique assignments compared to Maize2 having 26 shown in Fig. 4 (right). Only 1 GO assignment overlapped between Maize1 and Maize2.

### Maize1 genes have more isoforms than their Maize2 homeologs

**Fig. 5:**
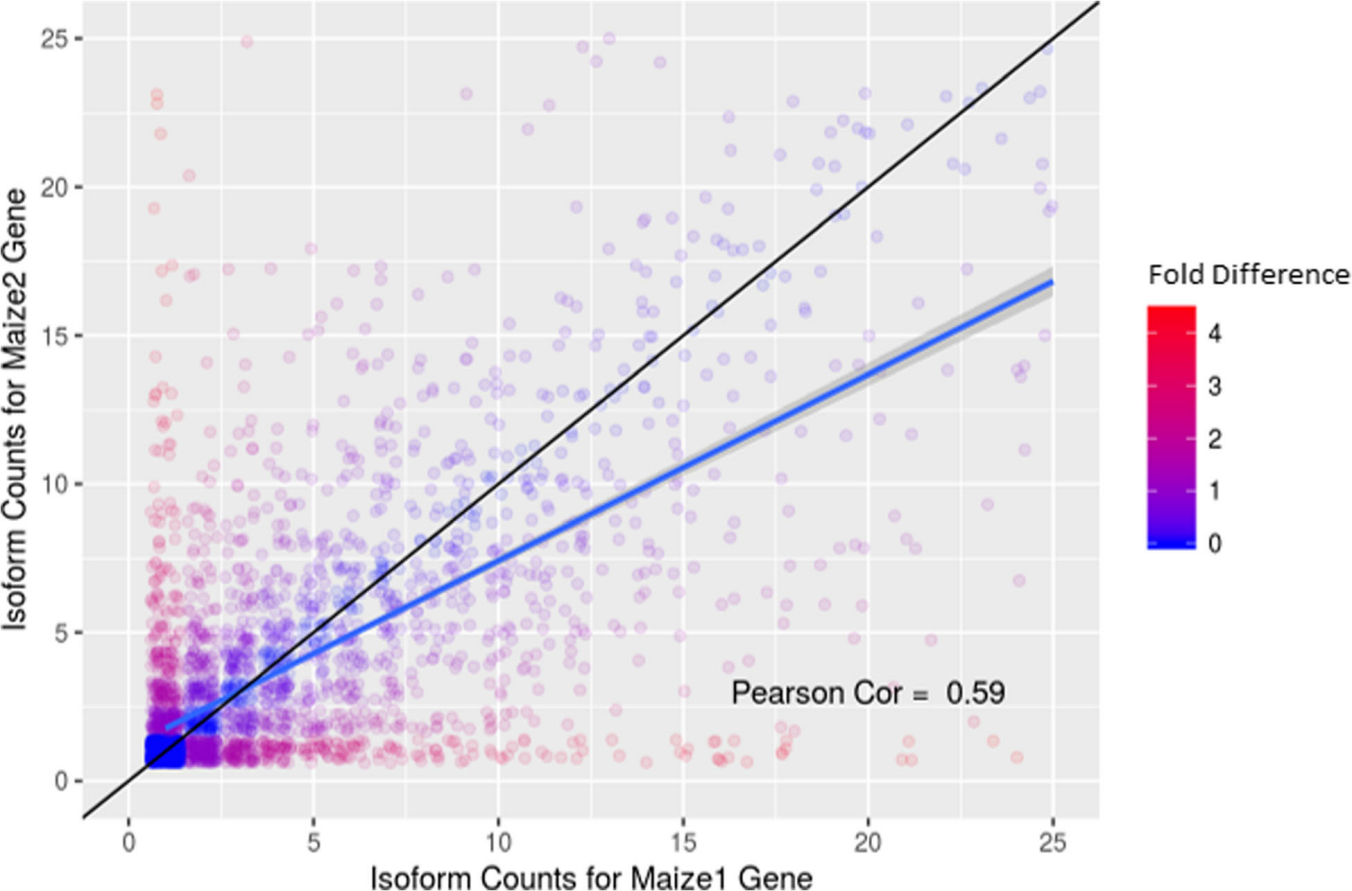
Number of isoforms for Maize1 homeolog vs. Maize2 homeolog plotted as a scatterplot. Black line represents a reference line for perfect fit. Blue line is the fit line. Maize1 tends to have more isoforms than Maize2.The jitter function of ggplot2 in R is used to spread overlapping points which are more highly concentrated at the lower numbers of isoforms per gene. The color scale reflects the log_2_ of the difference between the number of isoforms predicted for a Maize1 and Maize2 retained duplicate pair

Alternative splicing has been associated with multifunctional genes and changes to localization of gene products, thus an increase in unique isoforms should be indicative of a possible expansion in functions associated with a gene. We compared the number of isoforms predicted for both homeologs in each retained duplicate pair in Fig. 5. We find that genes in Maize1 are more likely to have a higher number of alternate splice forms than their Maize2 counterparts in the annotations that were provided by the B73 RefGen v4 assembly.

### Some reactions are catalyzed uniquely by the Maize1 and Maize2 subgenomes

A comparison between pathway and reaction associations in Maize1 with those in Maize2 show many reactions which are annotated in CornCyc uniquely to one subgenome. There are 687 reactions uniquely assigned to Maize1 and 255 uniquely assigned to Maize2. If we consider only retained duplicates genes and only reactions associated to at least one pathway, we find that Maize2 has more uniquely assigned reactions (see Additional file 4). The Maize1 subgenome catalyzes 19 reactions not available in Maize2, while Maize2 catalyzes 26 reactions not available in Maize1. Despite Maize2 enzymes catalyzing a larger set of unique reactions, these reactions represent a smaller group of pathways: Maize1 genes are involved in 14 pathways in which Maize2 is not directly contributing to, while Maize2 is only uniquely involved in 7 pathways.

## Discussion

**Fig. 6:**
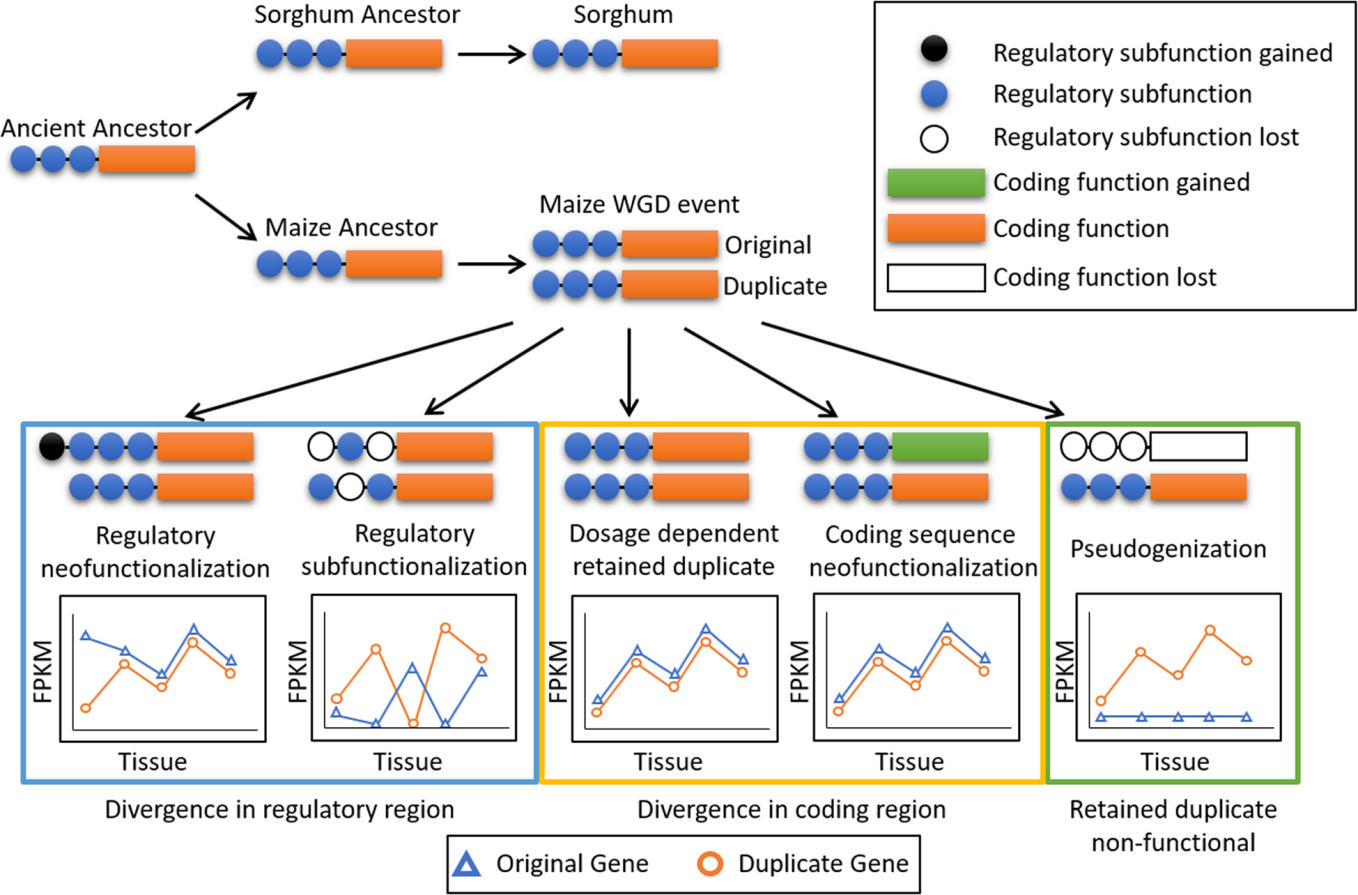
Sorghum and maize share a common ancient ancestor. After speciation, maize underwent a whole-genome duplication (WGD) event around 5–12 million years ago. Retained duplicates can be identified by synteny to the modern sorghum genome. As demonstrated in this figure, retained duplicates can evolve in several ways [14, 34, 35]

The expression and abundance patterns in maize retained duplicates viewed across a range of tissues provide insights into the evolutionary divergence of duplicated genes. The expression patterns depicted in Fig. 3 suggest how evolutionary pressures could change transcriptional regulation. Assuming retained homeologs were initially identical, this allows us to connect modern homeolog expression patterns to the neofunctionalization framework described in [33]. Sequence changes in the upstream region of a gene have the potential to alter the gene’s expression relative to its homeolog including gain or loss of regulatory subfunction or complete pseudogenization as shown in Fig. 6. A change in coding region would not be expected to affect expression patterns. Pseudogenization represents a case of fractionation as described previously by [15, 36].

The default assumption for a retained gene is that the expression patterns should be very similar. Indeed, this is the case for many of the putative retained duplicates which co-express to a high degree (based on correlation) and at similar levels. It is also possible for these types of retained duplicates to co-express with a high degree of correlation, but to have one of the genes express at a much lower level. Dosage sensitive genes were found to be retained at a higher rate after whole-genome duplication (WGD) events [37].

An interesting scenario that is not often discussed are cases were the dominant retained duplicate switch in different tissues. These cases are of interest as they imply that 1) both genes are expressed and are likely to have functional products, and 2) their dominance pattern changes and thus they are likely under different regulatory control. While this does not preclude the possibility that both genes have the same molecular function, it strongly implies that they evolved under separate evolutionary pressures and thus are more likely to have diverged in function.

Studies often find only moderate correlation (often between 40 and 50%) between gene expression measures and protein abundance measures when aggregated genome-wide [18, 38–41]*.* Some studies have found the correlation to be greater for differentially expressed genes [42]. We report a similar degree of correlation between gene and protein abundance values (Spearman correlation = 0.549). We find that the protein abundances are greater in Maize1 than in Maize2 when averaged across all tissues. There were more retained duplicates with detected non-zero protein abundance than gene expression, despite there being more genes across the whole genome overall with detected non-zero expression. In particular, we have narrowed down a small list of genes with similarly patterned expression and abundance, although we found that genes which are found to have no detectable expression often cannot be said to have no detectable protein (see Additional file 2).

Due to the difficulty of obtaining protein abundance data, expression data is often used in scientific studies, which raises the question of whether expression data correlate with protein abundance. Because mRNA levels are often transient, context sensitive, and may not affect phenotype due to post-translational regulation, protein abundance levels are often a more reliable indicator of genes affecting phenotype. Indeed, our results suggest that using protein abundance data can in fact provide additional insights, as several genes with little or no detected expression had in fact detectable protein abundance.

Retained duplicates not only vary in expression and abundance patterns across tissues, they also differ in functional annotations. We report that computationally assigned terms show a large discrepancy between the subgenomes. Since both homeologs are retained duplicates and were found to have similar syntenic regions, we had expected a greater overlap between their annotations. Part of the reason for this may be that synteny/orthology to other organisms and sequence similarity are often only one of the criteria for computationally assigning GO terms. This result suggests either 1) the homeologs are not similar enough for the computational algorithms to apply the same homology transfer to both homeologs (i.e. they have diverged) or 2) that the computational methods used to perform homology transfer are not set up to handle cases involving retained duplicates (i.e. they enforce a 1-to-1 match in orthology) in which case these GO terms should be viewed skeptically, or 3) gene loss in one or both subgenomes has left the other subgenome with unique functions.

Experimentally derived annotations also favor Maize1. While it is possible that this result is capturing some bias in literature indicating that the genes in Maize1 are more likely to be annotated than those in Maize2, taken at face value, given that some functional annotations are easier to be obtained than the others, this result would seem to reinforce the hypothesis that the Maize1 and Maize2 subgenomes have diverged functionally.

One working hypothesis on the evolutionary cause of biased fractionation is that the maize whole genome duplication event is thought to be the result of an allopolyploidization. It has been theorized that the parents going into an allopolyploid event in *Brassica rapa* [11] and other species had differing levels of transposon load, and this could have been true for the maize allopolyploidy event too. Since TE silencing can spread into genic regions [43], they may cause depletion in gene expression such that the separate lineages going into the polyploidy event had differing levels of whole-genome RNA expression [44]. The less-expressed subgenome is under less selection pressure [45], thus accumulating more deletions. As a consequence, the less fractionated subgenome (which we arbitrarily assign to Maize1) is expected to be the higher-expressed subgenome. This hypothesis is supported by the fact that known or predicted autopolyploids don’t seem to show evidence of fractionation bias [46].

## Conclusion

A comparison of expression and abundance patterns between retained duplicates in maize reveals evidence of functional divergence between homeologs consistent with the neofunctionalization framework, where the function of the original gene remains the same and the duplicated gene acquires a new function through neutral mutations [33]. Interestingly, pseudogenization does not appear to be the dominant form of divergence as there are relatively few pairs for which one of the genes in the pair did not show expression, and even fewer pairs for which one gene also did not have any protein abundance evidence. This suggests that homeologous pairs in maize continue to provide a selective advantage. The prevalence of tissue-specific alternating dominance patterns suggests that this advantage is related to a functional divergence. Differences in both manually and computationally assigned GO term annotations may reflect the divergence of one or both copies from their ancestral function.

Previous studies found a greater number of genes in Maize1 express higher than their Maize2 homeologs on average. Using updated definitions for the subgenomes on the current version of the maize reference genome, we find gene expression over a broad range of tissues also shows this bias towards greater expression of Maize1 genes. We find Maize1 has a greater range of GO annotations but were surprised to see that Maize2 is predicted to catalyze more unique reactions than Maize1. It is possible that fractionation bias which has been observed to cause greater gene loss in Maize2 translates to a greater likelihood that Maize2 genes will evolve new functions compared to genes in Maize1. We observe that protein abundance values for retained duplicates follow patterns similar to tissue-specific expression for the maize subgenomes. Differences in abundance patterns between homeologs can provide supporting evidence of functional divergence when considered alongside gene expression.

## Methods

### *Zea mays* genome sequence

The *Zea mays* genome sequence was retrieved from (ftp://ftp.ensemblgenomes.org/pub/release-37/plants/fasta/zea_mays/dna/Zea_mays.AGPv4.dna.toplevel.fa.gz) and the gene models from (ftp://ftp.ensemblgenomes.org/pub/release-37/plants/gtf/zea_mays/Zea_mays.AGPv4.37.gtf.gz).

### RNAseq expression and protein abundance datasets

A publicly available expression and protein abundance dataset described by Walley et al. [18] was used for this project. This dataset consists of both Illumina HiSeq2500 single strand reads and protein abundances collected concurrently for 23 tissues. The short read files were downloaded from GenBank’s Sequence Read Archive (SRA) [47] in Fastq format [48]. The protein data was projected from RefGen_v2 to RefGen_v4 using mapping files available at MaizeGDB. In cases were a gene model was deemed to have split into two or more new gene models, the dNSAF value of the RefGen_v2 gene model was assigned to each RefGen_v4 gene model. In cases where two or more gene models were deemed to have merged, the sum of the dNSAF values for each RefGen_v2 gene models was assigned to the RefGen_v4 gene model. (Please see Additional file 5 for expression data).

### Align RNAseq data to RefGen_v4

The expression data was aligned to the B73 RefGen_v4 assembly using STAR [29] version 2.5.2b. FastQC [49] revealed adapter contamination which was removed with Trimmomatic [50] version 0.36 using the following parameters: SE -phred33 ILLUMINACLIP: TruSeq3-SE.fa:2:30:10 TRAILING:3 MINLEN:36. The indexing step was done against the AGPv4 sequence guided by the AGPv4.37 gene models using the following parameters: --runMode genomeGenerate --sjdbOverhang 100. All other parameters were left default. Alignment of the fastq formatted reads against the index created in the previous step was done with the following parameters: --runMode alignReads --quantMode GeneCounts. All other parameters were left default. Cufflinks [30] version 2.2.1 was used to quantify gene expression in FPKM using the parameter --library-type fr-firststrand. FPKM was averaged across biological replicates for all tissues. STAR reported 4% of reads mapping to multiple loci. Cufflinks by default distributes multimapping reads uniformly to all positions that such reads can be mapped to. Please see Additional file 6 and Additional file 7 for details.

## Supplementary information

**Additional file 1.** Subgenome assignments in v4 for all genes, list of retained duplicate pairs, list of reactions associated with subgenome genes for all reactions and for the subset of reactions associated with at least one CornCyc pathway.

**Additional file 2.** Subgenome dominance in terms of expression and protein abundance for retained duplicate pairs.

**Additional file 3.** GO term assignment data.

**Additional file 4.** Pathway diagram of reactions unique to each subgenome.

**Additional file 5.** Expression data aligned to v4.

**Additional file 6.** STAR Aligner output showing summary of results across all tissue samples.

**Additional file 7.** Expanded description of methods, software, and parameters used in this study.

## Data Availability

This software and data used in this study are available at https://github.com/jrwalsh/SubGenome.

## Abbreviations

DAP: Days after planting
dNSAF: Distributed Normalized Spectral Abundance Factor
FPKM: Fragments per kilobase of transcript per million
GO: Gene ontology
INDELs: Insertions/deletions
Ks: Synonymous mutation rate
RNA: Ribonucleic acid
SNPs: Single nucleotide polymorphisms
SRA: Sequence Read Archive
TE: Transposable elements
WGD: Whole-genome duplication
