# Supplementary material for: Tissue-specific gene expression and protein abundance patterns are associated with fractionation bias in maize
Source: BMC Plant Biol. 2020 Jan 3;20:4. doi: 10.1186/s12870-019-2218-8 (PMC6942271; doi:10.1186/s12870-019-2218-8)
Supplement: Supplementary file 4 — Additional file 4. Pathway diagram of reactions unique to each subgenome. [file 12870_2019_2218_MOESM4_ESM.docx]

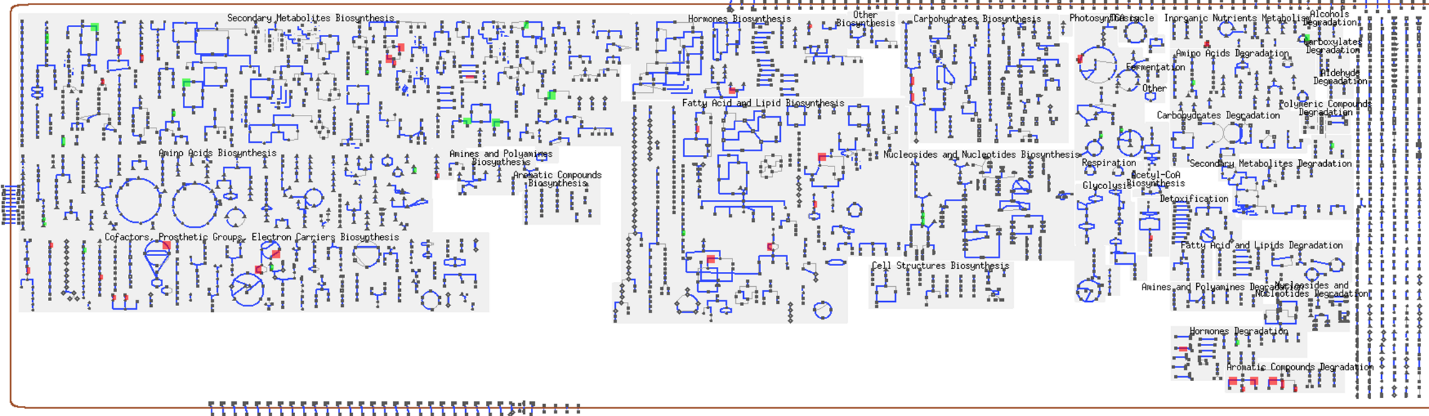


*Non-overlapping reactions associated with genes in each subgenome for retained pairs. Reactions predicted to be catalyzed by genes in subgenome. Red = Maize1 and Green = Maize2. Overlapping reactions catalyzed by both Maize1 and Maize2 are not highlighted. Not all available reactions are shown. The list of assigned reactions can be found in the Supplementary Materials S2.*
