# Supplementary material for: Tissue-specific gene expression and protein abundance patterns are associated with fractionation bias in maize
Source: BMC Plant Biol. 2020 Jan 3;20:4. doi: 10.1186/s12870-019-2218-8 (PMC6942271; doi:10.1186/s12870-019-2218-8)
Supplement: Supplementary file 7 — Additional file 7. Expanded description of methods, software, and parameters used in this study. [file 12870_2019_2218_MOESM7_ESM.docx]

# Subgenome Assignment

Synonymous mutation (Ks) rate cutoff of 1.0 was chosen based on Figure S1 center to differentiate between the most recent alpha duplication event and the previous beta duplication. By transforming the raw Ks value frequency (Figure S1 left) to the block median Ks values, we see two distinct peaks (Figure S1 center).


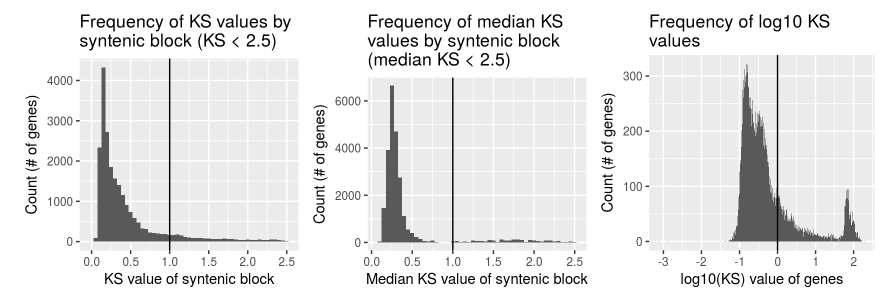


Figure S1: Ks values produced by SynMap. Left: KS values, showing only those < 2.5. Center: Ks values for each gene replaced with the median of the Ks values in the syntenic block, showing only values below 2.5. Right: Log10 of Ks values for all genes. Vertical line in each figure represents the arbitrarily chosen cutoff to separate the whole genome duplication event from older and/or more isolated duplications.

25 syntenic regions were found between maize and sorghum. To separate subgenome 1 from subgenome 2, we utilized a greedy approach to collect non-overlapping (when projected on sorghum) syntenic blocks by size, assigning the larger block to the Maize1 subgenome.

# Align RNAseq data to RefGen_v4

For this study, an expression atlas previously described in [1] was aligned to the AGPv4.0 B73 v4 gene model in order to study expression diversity between subgenomes. This dataset consists of 68 biological replicates over 23 tissues (3 reps per tissue with one tissue only having 2 reps) sequenced using Illumina HiSeq2500 single strand reads.

Fastq [2] formatted data was downloaded using SRAToolkit [3] for the SRA accessions listed here (<https://www.ncbi.nlm.nih.gov/Traces/study/?acc=PRJNA217053>). The Zea mays genome sequence was retrieved from (ftp://ftp.ensemblgenomes.org/pub/release-37/plants/fasta/zea_mays/dna/Zea_mays.AGPv4.dna.toplevel.fa.gz) and the gene models from (<ftp://ftp.ensemblgenomes.org/pub/release-37/plants/gtf/zea_mays/Zea_mays.AGPv4.37.gtf.gz>).

STAR [4] was used to perform the alignment. The indexing step was done against the AGPv4 sequence guided by the AGPv4.37 gene models using the following parameters: --runMode genomeGenerate --sjdbOverhang 100. All other parameters were left default. Alignment of the fastq formatted reads against the index created in the previous step was done with the following parameters: --runMode alignReads --quantMode GeneCounts. All other parameters were left default. Alignment resulted in an average 77% of reads mapped uniquely to the genome. Cufflinks [5] was used to quantify gene expression in FPKM using the parameter --library-type fr-firststrand. FPKM was averaged over bioreps for all tissues.

# Comparison of newly aligned RNAseq data to old alignment


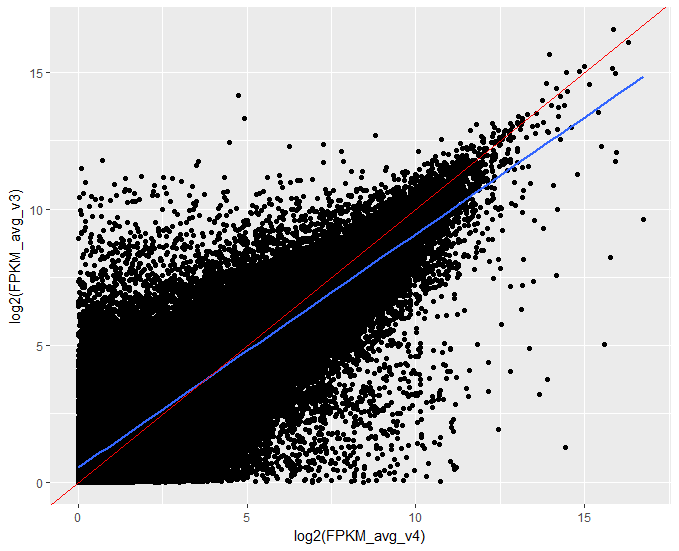


Figure 2: Scatterplot of FPKM (avg across replicates) for v3 vs v4 mappings on log2 scale – red line is reference, blue is lm fit.

# Calculating Syntenic Regions using SynMap

This analysis can be regenerated by following this link: <https://genomevolution.org/r/on51>.
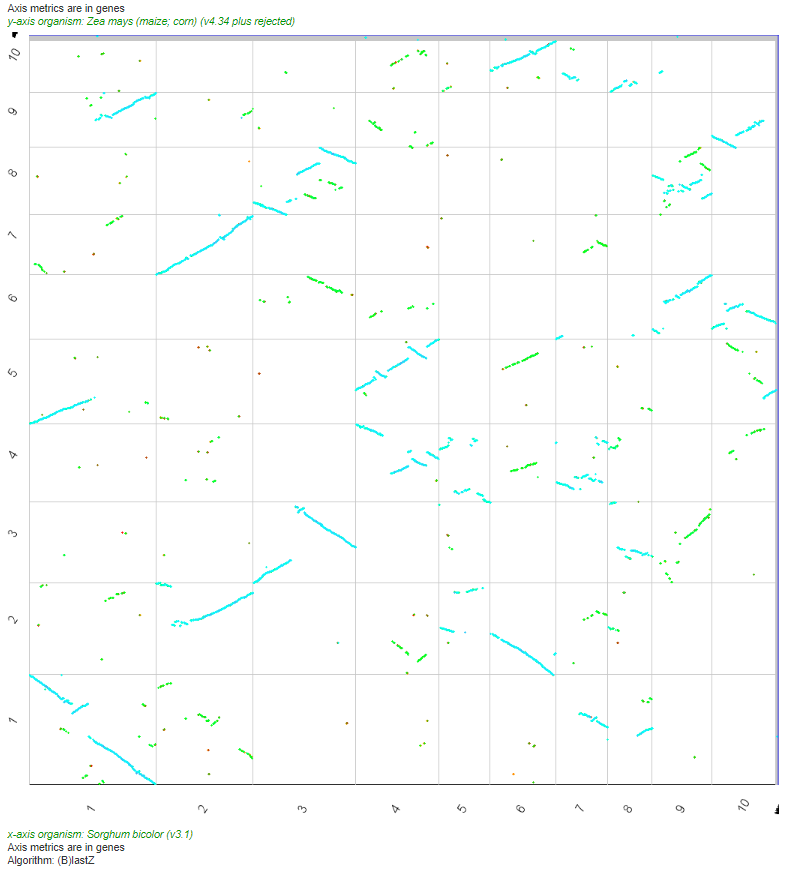


Figure 3: Syntenic regions between maize and sorghum. Light blue regions represent the most recent whole genome duplication event used as the basis for assigning genes to the maize1 and maize2 subgenomes.


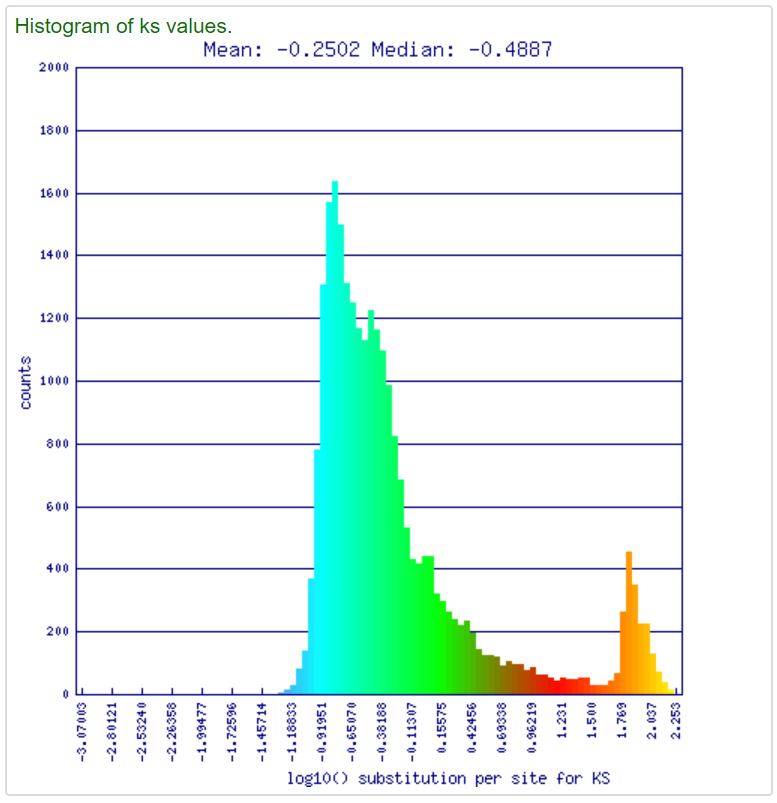


Figure 4: Duplicated genes with Ks values represented with green, red, orange, or yellow dots represent earlier duplication events outside the scope of this paper.

# Comparing Expression and Abundance Data

## Expression of Maize1 vs. Expression of Maize2 for Gene Pairs


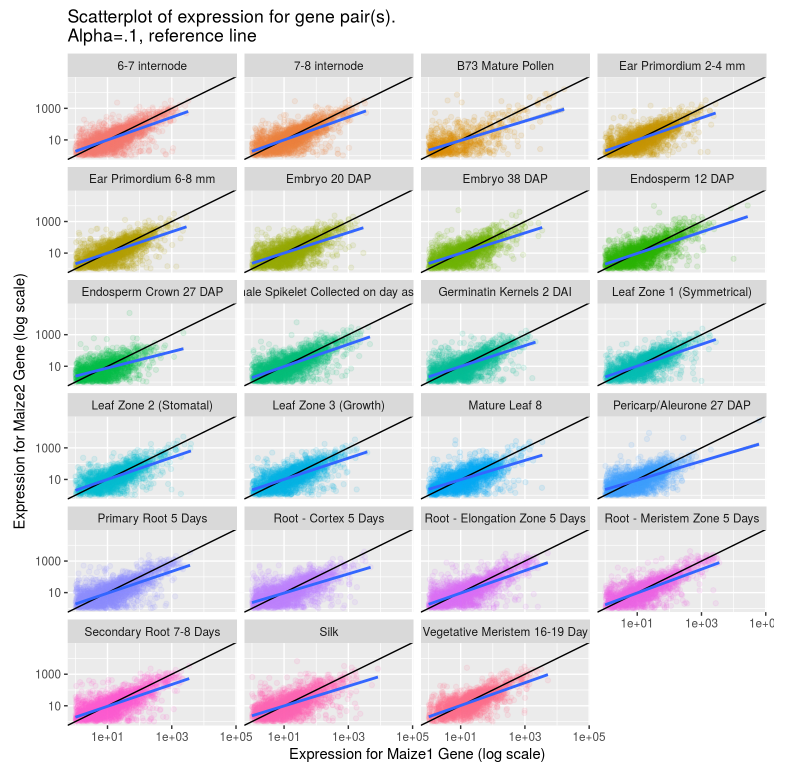


Figure 5: Scatterplot matrix for RNA expression of the Maize1 homeolog vs. the Maize2 homeolog in each of the 23 tissues measured in this dataset. Black line represents a reference line for perfect fit. Most tissues show modest positive correlation between expression of Maize1 and Maize2 homeolog. Expression values are shown as log2 of the FPKM for each gene. Log transformed expression helps visualize expression data, which tends to have most of the data in a very low range with some very extreme outliers. Using log2 makes fold change more apparent.

## Abundance of Maize1 vs. Abundance of Maize2 for Proteins of Gene Pairs


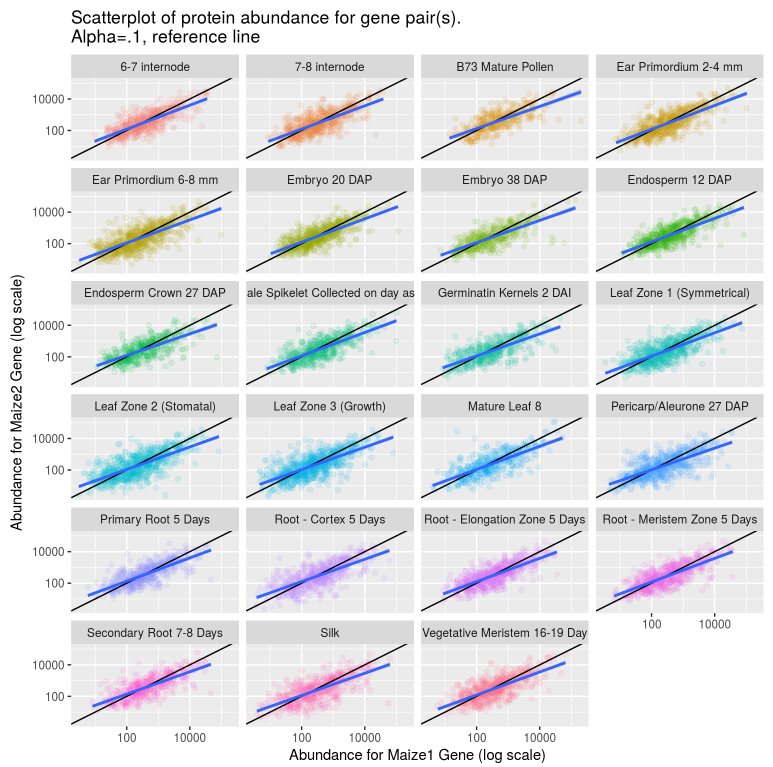


Figure 6: Scatterplot matrix for protein abundance of the Maize1 homeolog vs. the Maize2 homeolog in each of the 23 tissues measured in this dataset. Black line represents a reference line for perfect fit. Most tissues show modest positive correlation between abundance of Maize1 and Maize2 homeolog.

## Expression vs. Abundance for Genes in Maize1 Show Similar Patterns for Most Tissues


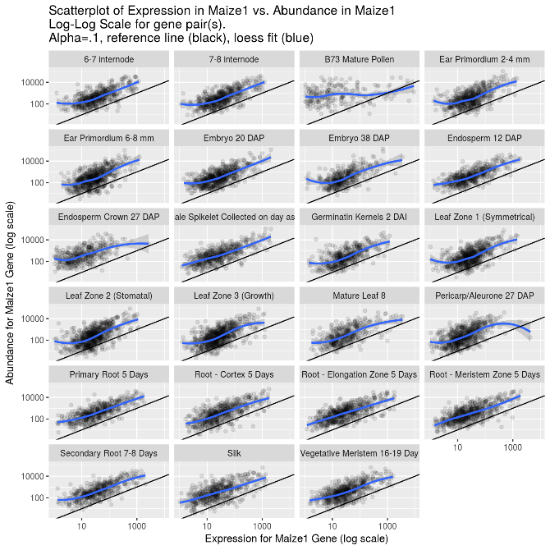


Figure 7: Scatterplot matrix for expression of the Maize1 homeolog vs. protein abundance of the Maize1 homeolog in each of the 23 tissues measured in this dataset. Black line represents a reference line for perfect fit. Blue line represents the fit line. Most tissues show weak positive correlation between expression and abundance. Note that Mature Pollen shows almost no correlation between expression and abundance. It is not known at this time if this is related to pollen being haploid.

## Change in Exp vs. Change in Abundance


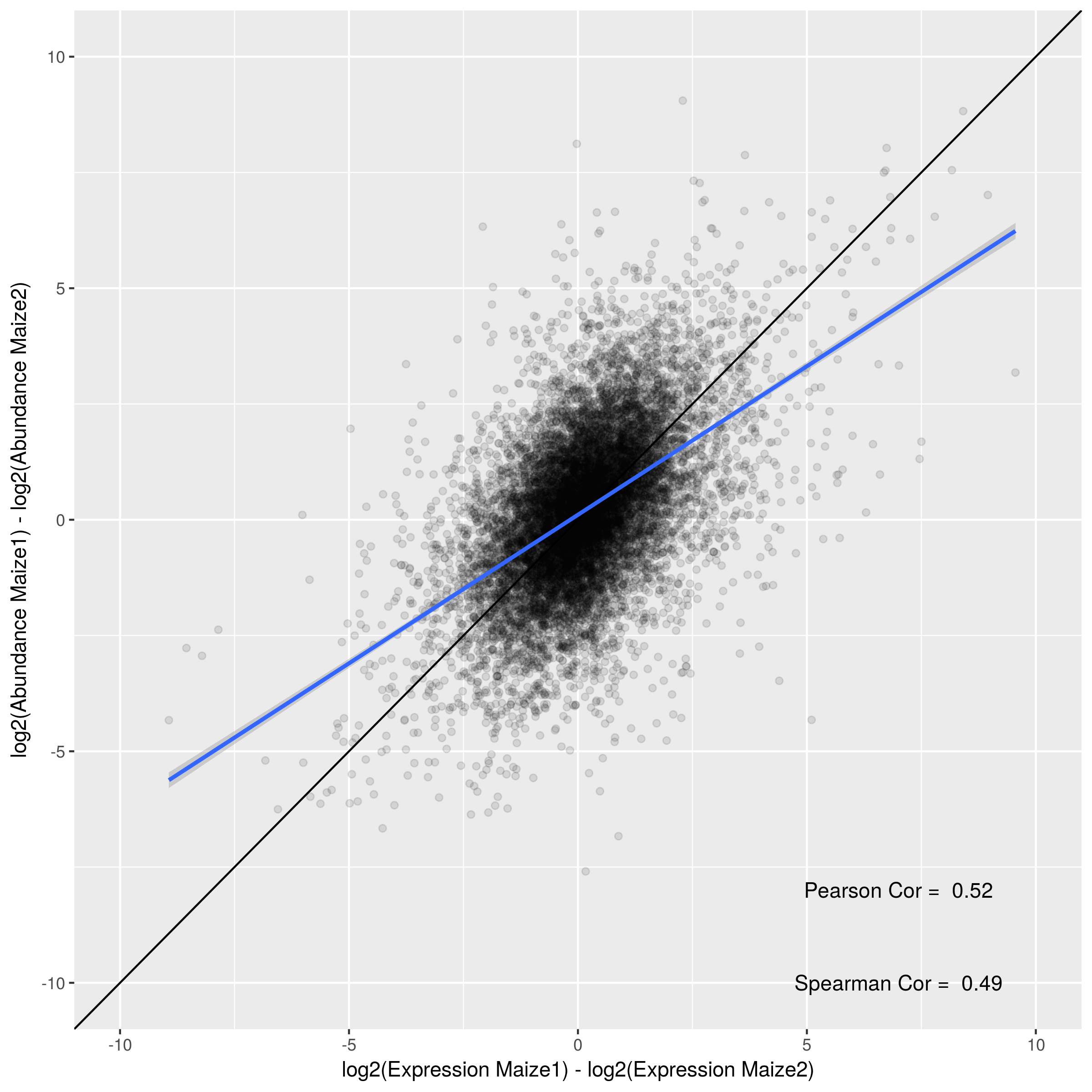


Figure 8: Considering only homoeologous pairs where both genes were measured for expression and protein abundance, we look at fold change from Maize2 expression to Maize1 expression vs. the fold change from Maize2 abundance vs. Maize2 abundance. A slightly positive trend indicates a mild positive correlation between an increase in expression and an increase in abundance.

# References

[1] J. W. Walley *et al.*, “Integration of omic networks in a developmental atlas of maize,” *Science*, vol. 353, no. 6301, pp. 814–818, Aug. 2016.

[2] P. J. A. Cock, C. J. Fields, N. Goto, M. L. Heuer, and P. M. Rice, “The Sanger FASTQ file format for sequences with quality scores, and the Solexa/Illumina FASTQ variants,” *Nucleic Acids Res.*, vol. 38, no. 6, pp. 1767–1771, Apr. 2010.

[3] R. Leinonen, H. Sugawara, and M. Shumway, “The Sequence Read Archive,” *Nucleic Acids Res.*, vol. 39, no. Database issue, pp. D19–D21, Jan. 2011.

[4] A. Dobin *et al.*, “STAR: ultrafast universal RNA-seq aligner,” *Bioinformatics*, vol. 29, no. 1, pp. 15–21, Jan. 2013.

[5] C. Trapnell *et al.*, “Differential gene and transcript expression analysis of RNA-seq experiments with TopHat and Cufflinks,” *Nat. Protoc.*, vol. 7, no. 3, p. 562, Mar. 2012.
